# A Molecular Survey of *Campylobacter jejuni* and *Campylobacter Coli *Virulence and Diversity

**DOI:** 10.6091/ibj.1359.2014

**Published:** 2014-07

**Authors:** Mahdi Ghorbanalizadgan, Bita Bakhshi, Anoshirvan Kazemnejad Lili, Shahin Najar-Peerayeh, Bahram Nikmanesh

**Affiliations:** 1 *Dept. of Bacteriology, Faculty of Medical Sciences, Tarbiat Modares University, Tehran, Iran; *; 2 *Dept. of Biostatistics, Faculty of Medical Sciences, Tarbiat Modares University, Tehran, Iran; *; 3 *Children’s Hospital Medical Center, Tehran university of Medical Sciences, Tehran, Iran*

**Keywords:** Campylobacter *jejuni*, *Campylobacter* coli, Ddiarrhea, Virulence factors

## Abstract

**Background**: The aim of this study was to determine the prevalence of virulence-associated genes and enterobacterial repetitive intergenic consensus PCR (ERIC-PCR) analysis of *Campylobacter *spp. isolated from children with diarrhea in Iran. **Methods:** A total of 200 stool specimens were obtained from children under 5 years during July 2012 to July 2013. Detection of *C. jejuni* and *C. coli* was performed by standard biochemical and molecular methods. The presence of virulence-associated genes and genetic diversity of isolates was examined using PCR and ERIC-PCR analyses. **Results:** A total of 12 (6%) *Campylobacter* spp. were isolated from patients including 10 (4.5%) *C. jejuni* and 2 (1.5%) *C.coli*. The *fla*A, *cad*F and *cia*B genes were present in 100% of isolates, while no plasmid of *vir*B11 gene was present in their genome. The prevalence of invasion-associated marker was 100% among* C. coli* and was not detected in* C. jejuni* isolates. The distribution of both *pld*A and the genes associated with cytolethal distending toxin (CDT) was 58.3% in *C. jejuni* isolates. Seven distinct ERIC-PCR profiles were distinguished in three clusters using ERIC-PCR analysis. Genotyping analysis showed a relative correlation with geographic location of patients and virulence gene content of isolates. **Conclusion:** To our knowledge, this is the first molecular survey of *Campylobacter* spp. in Iran concerning genotyping and virulence gene content of both *C. jejuni* and *C. coli*. ERIC-PCR revealed appropriate discriminatory power for clustering *C. jejuni* isolates with identical virulence gene content. However, more studies are needed to clearly understand the pathogenesis properties of specific genotypes.

## INTRODUCTION

*Campylobacter *spp. especially *C. jejuni and C. coli *are considered as potential etiological agents that caused many undiagnosed cases of acute diarrhea in children in developing countries including Iran [1-3]. The purpose of our experiments was to determine the rate and molecular survey of *C. jejuni* and *C. coli* virulence and also their diversity that leads to the practical applications to elucidate* Campylobacter* colonization and the control of this organism. Recurrent exposure to these organisms might raise level of specific immunity correlated with age in developing countries. Therefore, children younger than 5 years of age are mainly affected by these organisms [4]. 

Above organisms are fastidious and need nutrient-rich-based medium and microaerobic atmosphere [5]. This reason may be the main cause that *Campylobacter *spp. are not applied in routine diagnostic programs of clinical laboratories in most developing countries. The pathogenicity of *Campylobacter *species is dependent on their ability to bind to the human intestinal cells and *Cad*F protein. This protein, which is encoded by *cad*F gene, is responsible for *Campylobacter *binding to extracellular matrix of human intestinal cells [6]. Another gene, *fla*A, encodes a flagella protein which mediates motility, colonization, and invasion of gastrointestinal tract and it is essential for establishing human infection. 

The *cia*B (an invasion protein), *virB11 *(the IV secretory system), and *pld*A (an outer membrane phospholipase A) genes encode proteins associated with increased bacterial invasion on cultured epithelial cells; however, their exact roles in invasion have remained to be elucidated [7]. Cytolethal distending toxin (CDT) is encoded by three linked genes, including *cdt*A, *cdt*B, and *cdt*C. In epidemiology of infectious diseases, bacterial typing is of great value in source tracking studies. To analyze the genetic relatedness of *C. jejuni*, several molecular typing methods based on PCR have been developed. enterobacterial repetitive intergenic consensus PCR (ERIC-PCR) has been shown high discriminatory power, good legibility, and ease of use in most epidemiological investigations. 

In this study, we aimed to determine the prevalence of virulence-associated genes and ERIC-PCR analysis of *C. jejuni *and *C. coli *isolated from children with diarrhea. CDT is one of the most characterized virulence factors in C*ampylobacter *spp. Pathogenesis induces cell cycle arrest in G2 phase and promotes DNA damage together with apoptotic death in human monocytic cells; therefore, its presence is supposed to be associated with the severity of the disease.

## MATERIALS AND METHODS

***Study design and data collection procedure. ***The study was started after obtaining ethical approval from Research and Publication ethics office of Tarbiat Modares University (Tehran, Iran). A total of 200 stool specimens were collected from children with acute diarrhea attended to two major Children’s Hospital Medical Center in Tehran from July 2012 to July 2013. All children under five years of age were included in this study. Children with persistent diarrhea or previous treatment with antibiotics in the last 5 days were excluded from the study. Acute diarrhea was defined as diarrhea which takes about 14 days or less. Demographic data were collected by a co-worker resident in hospital. All samples were transported to the laboratory in a modified Cary-Blair transport medium (5.00 g/L sodium chloride, 1.50 g/L sodium thioglycollate, 1.10 g/L disodium phosphate, and 0.09 g/L calcium chloride, pH 8.4 ± 0.2 at 25ºC( with reduced agar content (1.6 g/L). Identification of *C. jejuni *and *C. coli *was performed by the standard culture, Gram staining, and conventional biochemical tests and confirmed by molecular methods [8, 9]. 

***Confirmation of presumptive Campylobacter species by duplex-PCR. ***Samples were incubated at 42°C for 48-72 h in microaerophilic conditions onto modified charcoal cefoperazone deoxycholate agar medium (10 g/L meat extract, 10 g/L peptone, 5 g/L sodium chloride, 4 g/L bacteriological charcoal, 3 g/L casein hydrolysate, 1 g/L sodium deoxycholate, 0.25 g/L iron (II) sulfate, 0.25 g/L sodium pyruvate, and 15 g/L agar, pH 7.4 ± 0.2 at 25º) plus campylobacter CCDA selective supplement (cefoperazone 3,200 mg/L). DNA templates were extracted by boiling method [10]. Confirmation of *Campylobacter *spp. was performed by PCR amplification of *cad*F gene. A duple-PCR was applied for simultaneous detection of *hipO *and *asp *genes, specific to *C. jejuni *and *C. coli*, respectively [11]. PCR was performed in a 25-μl reaction mixture, containing 10 ng DNA template, 2.5 μl PCR buffer 10×, 200 μM dNTP, 5 mM MgCl_2_, 0.1 μM each primer, 1 unit of Taq DNA polymerase, and deionized water. The primer sequences and their designation are shown in Table 1. The *C. jejuni *ATCC 29428 and *C. coli *ATCC 43478 were used as reference strains. 

***Detection of virulence/invasion-associated genes. ***The presence of virulence/invasion-associated genes, including invasion-associated marker (*iam)*, *pldA*, and *ciaB*, (responsible for *Campylobacter *invasion and attachment), *vir*B11 (involved in *Campylobacter *virulence), and CDT were investigated using specific primers which specifically amplify within the coding region of each gene. The distribution of *fla*A gene (responsible for *Campylobacter *attachment) was examined by primers specifically designed according to the *fla*A locus sequence of *C. jejuni *strain (GenBank accession no. AF050186.1). Due to the species-allele-specification of CDT sequence, two separate primer pairs were used which were selected according to *cdt *locus sequence of *C. jejuni *and *C. coli *standard strains. 

***Enterobacterial repetitive intergenic consensus PCR. ***ERIC-PCR assay was performed according to the method introduced by Versalovic and colleagues [12]. The primer sequences, their designation, and amplification conditions are depicted in Table 1. ERIC- PCR amplification reactions were performed in a 25-μl reaction mixture, containing 10 ng genomic DNA, 2.5 μl reaction buffer 10×, 200 μM dNTP, 5 mM MgCl_2_, 0.2 μM each primer, and 1 unit Taq DNA polymerase. The reaction was placed in a DNA thermal cycler (Mini Bio-Rad, USA). ERIC-PCR patterns were analyzed based on the Dice similarity coefficient using GelClust software [13].

## RESULTS

The study population was made up of 200 children with acute diarrhea, including 110 (55%) male and 90 (45%) female with the mean age of 27.4 months (2.3 years). Among 200 stool samples, *Campylobacter *spp., including 10 (4.5%) *C. jejuni *and 2 (1.5%) *C. coli *were isolated from 12 (6%) samples. 

**Table 1:** Primers, PCR conditions, and respective references

**Primers**	**Sequence (5** ' **→ 3')**	**Target**	**PCR condition**			**Amplicon** **(bp)**	**References**
			**Denaturin**	**Annealin**	**Extension**		
cadFU cadFR	TTGAAGGTAATTTAGATATG CTAATACCTAAAGTTGAAAC	*cad*F	94^ο^C, 30 s	43^ο^C, 30 s	72^ο^C, 30 s	400	[11]
hipOU hipOR	GAAGAGGTTTGGGTGGTG AGCTAGCTTCGCATAATAACTTG	*hip*O	94^ο^C, 30 s	53^ο^C, 30 s	72^ο^C, 30 s	735	[11]
aspU aspR	GGTATGATTTCTACAAAGCGAG ATAAAAGACTATCGTCGCGTG	*asp*	94^ο^C, 30 s	53^ο^C, 30 s	72^ο^C, 30 s	500	[11]
flaAU flaAR	TTTCGTATTAACACAAATGGTGC CTGTAGTAATCTTAAAACATTTTG	*fla*A	94^ο^C, 45 s	46^ο^C, 45 s	72^ο^C, 60 s	1743	this study
cdtjAU cdtjAR	AGGACTTGAACCTACTTTTC AGGTGGAGTAGTTAAAAACC	*Cj*-*cdt*A	94^ο^C, 30 s	55^ο^C, 30 s	72^ο^C, 30 s	631	[31]
cdtj BU cdtjBR	ATCTTTTAACCTTGCTTTTGC GCAAGCATTAAAATCGCAGC	*Cj-cdt*B	94^ο^C, 30 s	56^ο^C, 30 s	72^ο^C, 30 s	714	[31]
cdtjCU cdtjCR	TTTAGCCTTTGCAACTCCTA AAGGGGTAGCAGCTGTTAA	*Cj-cdt*C	94^ο^C, 30 s	55^ο^C, 30 s	72^ο^C, 30 s	524	[31]
cdtCAU cdtCAR	ATTGCCAAGGCTAAAATCTC GATAAAGTCTCCAAAACTGC	*Cc-cdt*A	94^ο^C, 30 s	55^ο^C, 30 s	72^ο^C, 30 s	329	[31]
cdtCBU cdtCBR	TTTAATGTATTATTTGCCGC TCATTGCCTATGCGTATG	*Cc-cdt*B	94^ο^C, 30 s	56^ο^C, 30 s	72^ο^C, 30 s	413	[31]
cdtCCU cdtCCR	TAGGGATATGCACGCAAAAG GCTTAATACAGTTACGATAG	*Cc-cdt*C	94^ο^C, 30 s	55^ο^C, 30 s	72^ο^C, 30 s	313	[31]
ciaBU ciaBR	TGCTAGCCATACTTAGGCGTTTT TTGATAATAGCGGACAATTTGAAA	*cia*B	94^ο^C, 30 s	54^ο^C, 30 s	72^ο^C, 30 s	610	this study
pldAU pldAR	AAGCTTATGCGTTTTT TATAAGGCTTTCTCC	*Pld*A	94^ο^C, 30 s	46^ο^C, 30 s	72^ο^C, 30 s	913	[32]
iamAU iamAR	GCGCAAAATATTATCACCC TTCACGACTACTATGCGG	*iam*	94^ο^C, 30 s	47^ο^C, 30 s	72^ο^C, 30 s	518	[33]
virB11U virB11R	GAACAGGAAGTGGAAAAACTAGC TCCCGCATTGGGCTATATG	*vir*B11	94^ο^C, 30 s	52^ο^C, 30 s	72^ο^C, 120 s	708	[33]
ERICF ERICR	ATGTAAGCTCCTGGGGATTCA AAGTAAGTGACTGGGTGAGCG	ERIC	94^ο^C, 30 s	52^ο^C, 30 s	72^ο^C, 300 s	variable	[34]

**Fig. 1:**
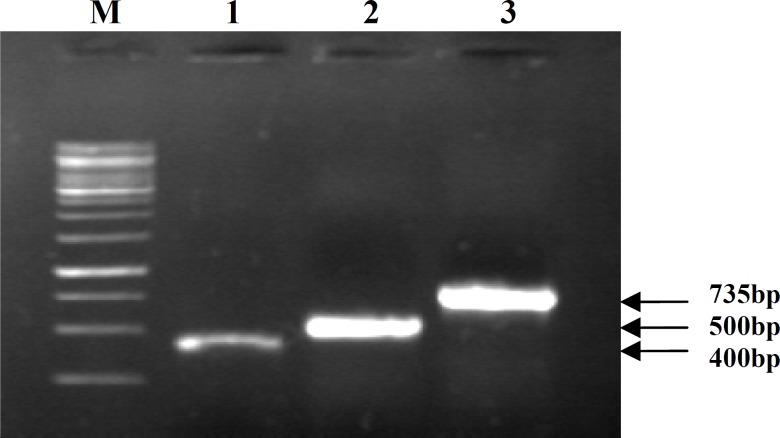
PCR and Duplex- PCRfor species identification of *Campylobacter* spp. and *C*. *jejuni/ C.coli* isolates, respectively; Lane1,* cad*F gene; lane 2, *asp* gene; lane 3, *hip*O gene, and M, 1 kb DNA size marker

***Duplex-PCR assay.*** The *cadF *gene was positive in 12 (100%) suspected cultures, and an amplification band of 400 bp was obtained for *cadF+ *isolates indicative of *Campylobacter *spp. Duplex-PCR for species identification indicated the presence of 10 (4.5%) *C*. *jejuni *and 2 (1.5%) *C. coli *isolates, respectively (Fig. 1).

***Prevalence of virulence-associated genes and cytolethal distending toxin.*** All of our *Campylobacter *isolates harbored *fla*A and *cia*B genes. CDT encoding genes (*cdt*A, *cdt*B, and *cdt*C) involved in CDT production, and *pld*A was found in 7 (58.3%) of *C. jejuni *isolates corresponding to amplification bands of 631, 714, 524, and 913 bp, respectively. However, no CDT encoding genes were found among *C. coli *isolates. The plasmid *vir*B11 gene was also absent among our *Campylobacter *spp. isolates. The prevalence of *iam *was 100% among *C. coli*, while no amplification was obtained for *C. jejuni *isolates (Table 2). 

**Fig. 2:**
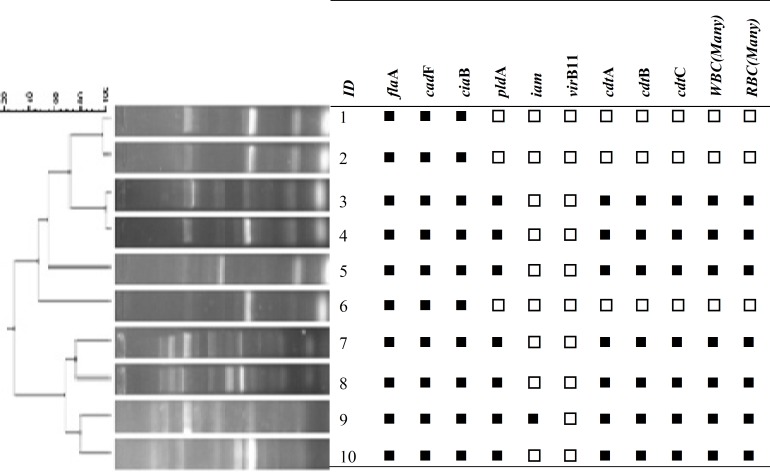
Dendrogram generated from the ERIC-PCR profiles of *C. jejuni* isolates from humans in relation to their Profile of virulence-associated genes. Strains 1, 2, 3, and 4 share 80% overall similarity, but strains 7, 8, 9, and 10 share 70% overall similarity.   positive   Negative

***Enterobacterial repetitive intergenic consensus PCR.*** In total, 10 out of 12 *Campylobacter *isolates under study produced 7 distinguishable banding profiles by ERIC-PCR genomic fingerprinting, which were corresponded to 10 *C. jejuni *isolates. Dendrogram of ERIC-PCR was created with UPGMA algorithm, which revealed 3 major clusters with 4, 4, and 2 members. No obvious banding pattern was obtained for *C. coli *isolates even after multiple attempts (Fig. 2). 

## DISCUSSION

Campylobacteriosis is one of the most common bacterial causes of food-borne illness and leading cause of bacterial diarrheal disease in the world [9]. In the present study, the prevalence of *Campylobacter *spp. among diarrheic children was about 6%. In a few previous studies performed in Iran, the prevalence of *Campylobacter *spp. was reported to be 8%, 10.8%, and 8.7% in 2007, 2009, and 2011, respectively [3, 14, 15]. This observation shows the trend of *Campylobacter *infections to be almost unchanged through years in this country. 

The prevalence of *Campylobacter *species among diarrheic children was reported to be 5.4% in Turkey [14], 7% in India [15] and 11.1% in Lebanon [16]. As reported by WHO [17], the incidence of Campylobacteriosis was 9.3 per 1,000 people in Europe America [17]. All isolates in the present study, either *C. Jejuni *or *C .coli*, harbored *cad*F, *fla*A, and c*ia*B genes. These genes are essential virulence factors involved in *Campylobacter *adhesion and colonization to human intestinal epithelial cells during human infection. The ubiquitous existence of the highly conserved *cad*F gene in 100% of *Campylobacter *spp. was previously reported by Konkel and coworkers [18] and was subsequently used by other investigators for successful detection of *Campylobacter *spp. [19, 20].

The prevalence of virulence-associated genes (*cia*B and *fla*A) was reported to be 80-100% in different studies concerning *Campylobacter *spp. infections in children with moderate to severe diarrhea [21-23]. Similarly, these genes were also detected within all of our isolates. The *cia*B and *fla*A are both involved in maximal invasion of human intestinal cells. This result can justify broadly existence of these gens in clinical *Campylobacter *spp. 

The *vir*B11 gene was not found in any of *Campylobacter *isolates under study. This finding is in agreement with the studies by other investigators who did not find* vir*B11 gene among *Campylobacter *isolates of children from Brazil and Bangladesh [23, 24]. However, a few other studies indicated the prevalence of *vir*B11 to be 10.7-22.7% among clinical isolates [21, 25]. This finding emphasizes the low prevalence of type IV secretion system apparatus in 

**Table 2:** Prevalence of virulence and toxin genes in *C.  jejuni* and *C. coli* isolates under study

**Species (No.)**	**No. of PCR positive (%)**										
	***cad*** ** F**	***hip*** **O**	***asp***	***vir*** **B11**	***cia*** **B**	***iam***	***pld*** **A**	***fla*** **A**	***cdt*** **A**	***cdtB***	***cdtC***
*C. jejuni* (10)	10 (100)	10 (100)	0 (0)	0 (0)	10 (100)	0 (0)	7 (58.3)	10 (100)	7 (58.3)	7 (58.3)	7 (58.3)
*C. coli* (2)	2 (100)	0 (0)	2 (100)	0 (0)	2 (100)	2 (100)	0 (0)	2 (100)	0 (0)	0 (0)	0 (0)
Total (12)	12 (100)	10 (83.4)	2 (16.6)	0 (0)	12 (100)	2 (16.6)	7 (58.3)	12 (100)	7 (58.3)	7 (58.3)	7 (58.3)

*Campylobacter *spp., which can probably be due to plasmid basis of the gene.

In this study, the *iam *gene that codes for *iam* was detected in 100% of *C. coli *isolates, while none of *C. jejuni *isolates harbored the gene. Similar results have been also reported regarding the high prevalence of *iam *gene among *C. coli *as well as its absence or low distribution among *C. jejuni *isolates of children with diarrhea in Brazil [26]. However, several studies demonstrated no substantial difference in its occurrence among the two species. This result shows that *iam *frequency is controversial [21, 27, 28]. 

The distribution of both *pld*A and the genes associated with CDT production (*cdt*A, *cdt*B, and *cdt*C) was 58.3% in *C. jejuni *isolates, while none of the genes were detected among *C. coli *isolates. The CDT toxin induces cell cycle arrest in G2 phase and promotes DNA damage; therefore, its presence is supposed to be associated with the severity of the disease in *C. jejuni*. However, variations which may occur within *cdt *gene sequences and may affect their detection through amplification methods should not be ignored during interpretation of negative results in *C. coli*. Moreover, a direct correlation was observed in this study between detection of *pld*A gene and the presence of white and red blood cells in stool of patients, which may be due to contribution of *pldA *gene product in pathological changes in intestinal epithelium. In agreement with our results, Rizal and colleagues [21] reported the presence of *cdt and pldA *genes among 50% and 55% of *C. jejuni *isolates, respectively, while none of the genes were detected within their *C. coli *isolates. Seven distinct ERIC-PCR profiles were distinguished from 10 *C. jejuni *isolates which were located in three clusters by ERIC-PCR. Cluster analysis showed that all isolates (no. 4) within cluster I were isolated from patients who lived in the south of Tehran. Except one isolate, all others within cluster II (no. 3) have also isolated from the patients in a similar geographical location in Tehran (east and center). Moreover, all of the isolates in cluster I and II revealed identical virulence gene content. Two isolates within cluster III were isolated from west of Tehran, but no clear correlation could be determined between ERIC-PCR profile and virulence genes content of isolates within this cluster. Sahilah and colleagues [29] reported that no specific relationship could be extracted between ERIC-PCR analysis and virulence gene content of their *C. jejuni *isolates. However, Wardak *et al*. [30] showed that ERIC-PCR could clearly divide *C. jejuni *and *C. coli *into two clusters. 

ERIC-PCR was unable to type our *C. coli *isolates which raises the question that to how extent is the typeability power of ERIC-PCR for *C. coli *strains. This emphasizes that more studies are needed to clearly understand the ability and role of this typing method in *C. coli *epidemiological studies. However, it is noteworthy that ERIC-PCR analysis has been proved as a well-documented molecular tool in epidemio-logical studies of *C. jejuni *strains. 

To our knowledge, this is the first molecular survey of *C. jejuni *and *C. Coli *genotypes in Iran. Nevertheless, further studies are needed to more clearly understand the correlation between virulence-associated genes and specific genotypes of *C. jejuni *and *C. coli *clinical isolates.
